# Forecasting Participants in the All Women Count! Mammography Program

**DOI:** 10.5888/pcd15.180177

**Published:** 2018-10-25

**Authors:** Calla Holzhauser, Patricia Da Rosa, Semhar Michael

**Affiliations:** 1 Mathematics and Statistics Department, South Dakota State University, Brookings, South Dakota; 2 Office of Research, College of Nursing, South Dakota State University, Brookings, South Dakota

## Abstract

**Introduction:**

The All Women Count! (AWC!) program is a no-cost breast and cervical cancer screening program for qualifying women in South Dakota. Our study aimed to identify counties with similar socioeconomic characteristics and to estimate the number of women who will use the program for the next 5 years.

**Methods:**

We used AWC! data and sociodemographic predictor variables (eg, poverty level [percentage of the population with an annual income at or below 200% of the Federal Poverty Level], median income) and a mixture of Gaussian regression time series models to perform clustering and forecasting simultaneously. Model selection was performed by using Bayesian information criterion (BIC). Forecasting of the predictor variables was done by using an autoregressive integrated moving average model.

**Results:**

By using BIC, we identified 5 clusters showing the groups of South Dakota counties with similar characteristics in terms of predictor variables and the number of participants. The mixture model identified groups of counties with increasing or decreasing trends in participation and forecast averages per cluster.

**Conclusion:**

The mixture of regression time series model used in this study allowed for the identification of similar counties and provided a forecasting model for future years. Although several predictors contributed to program participation, we believe our forecasting analysis by county may provide useful information to improve the implementation of the AWC! program by informing program managers on the expected number of participants in the next 5 years. This, in turn, will help in data-driven resource allocation.

**MEDSCAPE CME**

Medscape, LLC is pleased to provide online continuing medical education (CME) for this journal article, allowing clinicians the opportunity to earn CME credit.

In support of improving patient care, this activity has been planned and implemented by Medscape, LLC and *Preventing Chronic Disease*. Medscape, LLC is jointly accredited by the Accreditation Council for Continuing Medical Education (ACCME), the Accreditation Council for Pharmacy Education (ACPE), and the American Nurses Credentialing Center (ANCC), to provide continuing education for the healthcare team.

Medscape, LLC designates this Journal-based CME activity for a maximum of 1.00 *AMA PRA Category 1 Credit(s)™*. Physicians should claim only the credit commensurate with the extent of their participation in the activity.

All other clinicians completing this activity will be issued a certificate of participation. To participate in this journal CME activity: (1) review the learning objectives and author disclosures; (2) study the education content; (3) take the post-test with a 75% minimum passing score and complete the evaluation at http://www.medscape.org/journal/pcd; (4) view/print certificate.

**Release date: October 25, 2018; Expiration date: October 25, 2019**

**Learning Objectives**

Upon completion of this activity, participants will be able to:

1. Describe present usage of mammography screening in the AWC! program and clusters of counties with similar socioeconomic characteristics
2. Forecast future usage of mammography screening in the AWC! program
3. Determine public health implications of findings regarding present and forecasted usage of mammography screening in the AWC! program

**EDITOR**

Rosemarie Perrin

Editor, Preventing Chronic Disease

Disclosure Rosemarie Perrin has disclosed no relevant financial relationships.

**CME AUTHOR**

Laurie Barclay, MD

Freelance writer and reviewer, Medscape, LLC

Disclosure: Laurie Barclay, MD, has disclosed the following relevant financial relationships:

Owns stock, stock options, or bonds from: Pfizer Inc.

**AUTHORS**

Calla Holzhauser, MSc

Mathematics and Statistics Department, South Dakota State University, Brookings, South Dakota

Disclosure: Calla Holzhauser, MSc, has disclosed no relevant financial relationships.

Patricia Da Rosa, DDS, MSc

Office of Research, College of Nursing, South Dakota State University, Brookings, South Dakota

Disclosure: Patricia Da Rosa, DDS, MSc, has disclosed no relevant financial relationships.

Semhar Michael, PhD

Mathematics and Statistics Department, South Dakota State University, Brookings, South Dakota

Disclosure: Semhar Michael, PhD, has disclosed no relevant financial relationships.

## Introduction

An estimated 1 in 8 women will be diagnosed with breast cancer at some point in their lives (1). In 2014, breast cancer was the second leading cause of cancer death in women in South Dakota, and 608 women were newly diagnosed with the disease (2). More than half of the new breast cancers were diagnosed and reported at a localized (early) stage. Since 1997, South Dakota has administered mammograms and Papanicolaou (Pap) smears to women who qualify under the All Women Count! (AWC!) program (3). From 2012 through 2016, the AWC! program screened more than 3,914 eligible women for breast cancer (4).

AWC! is part of the National Breast and Cervical Cancer Early Detection Program (NBCCEDP), which has been the subject of ample research and reporting (5–8). *The Journal of Cancer Causes and Control* published an entire issue dedicated to the effects of NBCCEDP (7), and several studies have reported on the program, including the proportion of women reached and the program’s impact on breast cancer mortality rates among low-income women (annual incomes at or below 200% of the Federal Poverty Level). Other articles did not discuss NBCCEDP but discussed disparities in cancer screening among various groups (9,10) with some specific to breast cancer screenings (11–13). To our knowledge, however, forecasting participation in NBCCEDP has not been done. Forecasting participation in AWC!, an NBCCEDP program, would assist with planning and resource allocation and thereby increase access to timely breast cancer screening among underserved women in South Dakota. The goals of our project were to forecast the number of participants for South Dakota’s 66 counties and to identify county clusters within the state that share similar socioeconomic characteristics and that are rural with low populations. We fit a model within each cluster simultaneously by using a finite mixture of Gaussian regression time series models (14).

## Methods

### Data source

The AWC! data set consisted of patient sociodemographic information, residential information, date of visit to health care provider, and medical screenings from 1997 through 2017. Our analysis focused on breast cancer screening, both mammography and clinical breast examination (CBE). The data set did not include counts from other programs operating in South Dakota that offered free or reduced-cost mammograms, including those of the Indian Health Service. We counted only the first mammography visit per year. If a woman had abnormal results and required an additional mammogram, only the first mammogram was counted. Additional data sources were used to gather further predictors. Locations of participating mammography clinics in the state over a 10 year period (2005–2015) were also provided to us by the South Dakota Department of Health. We used the US Census Bureau’s Small Area Income and Poverty Estimates (SAIPE) database to obtain the median income of residents and the percentage of the population with annual incomes at or below 200% of the Federal Poverty Level (hereinafter poverty percentage) for each county (15) and the census bureau’s Population and Housing Unit Estimates database (16) for estimates by sex, race, and age group. We extracted the population of women aged 40 to 64 from the latter database at the county level by year and used this for our analysis.

The initial AWC! program screening data set contained 63,990 rows with 26,988 unique participants. At the time of our analysis, 2017 data were not complete and were removed, reducing our row count by 2,139 rows. Our analysis was concerned only with breast cancer screening (mammography and CBE). All participants who received either a CBE or a mammogram were kept. Next, we removed all participants from outside South Dakota, because our analysis included only South Dakota residents. Although women aged 30 to 39 are eligible under NBCCEDP to receive a CBE but not a mammogram, women in this age group were outside the scope of our study and were therefore excluded, reducing the data by 15,783 rows. Ninety-one percent of these of these removed rows contained data on women aged 30 to 39 who received only CBE. Our analysis was concerned only with the number of AWC! participants, defined as women aged 40 to 64 who received a CBE or mammogram at least once during a given year, regardless of their number of clinic visits in a given year. We then obtained counts of the number of participants per year by county. This yielded a total of 37,922 CBE or mammogram visits from 1997 through 2016. The SAIPE and Population and Housing Estimates data sets containing the 3 predictors (ie, population, poverty percentage, and median income) were joined to these counts on the basis of year and county.

### Statistical analysis

Some South Dakota counties are very rural and thus had a small number of participants. By grouping these counties together into clusters, we increased the amount of data used to build our forecasting model, thereby increasing the model’s robustness. The advantages of this were twofold. First, we could identify similar counties for future program modifications. Second, we took into account that the number of participants over the years for a given county were autocorrelated and not independent. These 2 procedures can be done simultaneously by using a finite mixture model of Gaussian regression time series. The model is given by

f(**y**_i_; **X**_i_, **Ψ) = **Σ_k_ τ_k_ ϕ_T_(**y**_i_; **X**_i _
**β**_k_, **Σ**_k_),

where *τ_k_*’s for *k=1,…,K *are mixing proportions and have the restrictions 0 *<*τ_k_ *≤* 1 and must sum to 1. *ϕ_T _* is a *T*-variate Gaussian distribution, and ***X*** *_i_* ***β*** *_k_, *and ***Σ*** *_k _* are the mean vector and covariance matrix of the Gaussian distribution. Therefore, we model ***y*** *_i_-* ***X*** *_i_* ***β*** *_k _* as a zero-mean autoregressive–moving average (ARMA) (*p, q*) time series, where ***y*** *_i_* is a *T*-variate response vector and ***X*** *_i_* is a *T × m* matrix of predictor variables, where *m* is the number of predictor variables in the regression model. The model parameters were estimated by using the Expectation Maximization algorithm (17). The result from the Expectation (E)-step, deals with identifying groups of similar counties that exist in the data and the Maximization (M)-step, provides the parameter estimates within each group identified from the E-step. These 2 steps are iterated until a convergence criterion is met indicating that the best solution was achieved. More details on this model are available (14). This mixture model is used to find similar counties and to build a single regression ARMA model within each cluster. In our work, the optimal number of clusters was determined by using the Bayesian information criterion (BIC) (18).

Once models were trained on the currently available data, forecasting was carried out. All the variables used as predictors in the models needed to be available for the forecast period. To accomplish this, we used a simple ARIMA (autoregressive integrated moving average) (*p, d, q*) model, which is an ARMA model, with I for Integrated, meaning *y_t _* differenced to create a stationary time series. The model is given by Φ(*B*)(1-* B*)*^d^y_t_* = (1 + *θ*(*B*))*ε_t_* where Φ(*B*) = 1 − ϕ_1_ *B* – *ϕ_2_B* ^2^ *−*…− *ϕ_p_B^p^*, θ(*B*) = θ_1_ *B* + … + *θ_q_B^q^*, and *B* is a backshift operator such that *B^j^* (*y_t_*) = *y_t_*_–_ *_j_*. *ε_t_* is assumed to be white noise. The optimal orders for this model, *p*, *q*, and *d*, were found by using the Akaike information criterion (19). This model was fitted by using the R package forecast (20). After obtaining the best model for each county and forecast predictors, we forecast the next 5-year counts. Model assessment was done through the validation set approach. The data set was split into training and validation by using year. The first 17 years of data were used for the training set, and the remaining 3 years were used for the validation set. The validation mean squared error (MSE) was calculated to assess the accuracy of our forecasting algorithm. The MSE was calculated as follows: *MSE = 1/n Σ_i_(Y_i_ - Ŷ_i_ )^2 ^.* We define *n* as the total number of forecasts, *Y_i_* as the observed count, and *Ŷ_i_* as the forecast for *i=1,…,n*. All analysis was completed using R version 3.4.2 (R Corporation).

## Results

**Figure 1:**
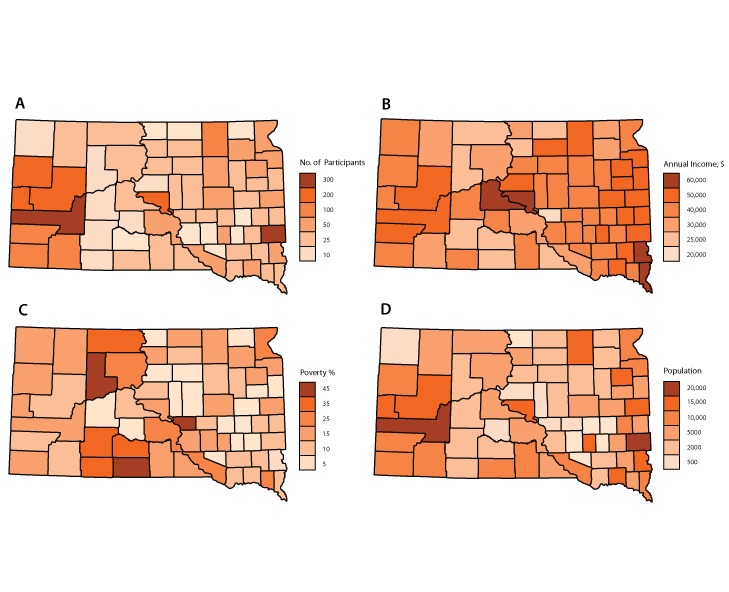
Average number of participants in the All Women Count! program (AWC!) by median income, poverty percentage (percentage of population with annual incomes at or below 200% of the Federal Poverty Level), and population for each South Dakota county,1997–2016.

The number of AWC! participants increased steadily from 1997 through 2011 and then sharply decreased in 2012. Since then, participation steadily decreased. Some counties had similar sociodemographic characteristics (average number of participants , median income, poverty percentage, and population) for the 1997–2016 time period (Figure 1). Minnehaha and Pennington were the most populated counties and had the largest number of AWC! participants (Table 1). Corson, Dewey, Buffalo, and Ziebach all had populations with a low median income and a high poverty percentage. Finally, Brown, Codington, and Lincoln had low poverty percentages and high populations. The mixture model described above was used and the optimal number of clusters was determined to be 5.

**Table 1:** All Women Count! Participants in South Dakota Counties by Demographic Characteristics and Population, 1997–2016

County	Average No. Participants	Average of US Census Median County Income	Average Poverty Percentage^a^	Average Population
Aurora	8.11	38,045.11	12.03	423.58
Beadle	31.79	38,372.68	12.76	2,729.05
Bennett	5.89	29,334.05	33.16	465.11
Bon Homme	15.89	37,045.16	14.22	970.74
Brookings	23.37	43,479.53	12.96	3,573.16
Brown	56.16	43,380.11	10.26	5,724.32
Brule	12.68	38,580.37	13.35	821.63
Buffalo	15.42	18,621.58	37.84	247.95
Butte	55.26	35,539.00	14.57	1,611.05
Campbell	5.16	35,874.68	11.68	264.68
Charles Mix	24.47	32,096.42	23.52	1,335.95
Clark	8	38,030.89	13.36	595.90
Clay	11.32	35,241.79	19.90	1,491.63
Codington	34.53	42,772.37	10.36	4,120.21
Corson	5.47	25,238.32	36.26	549.90
Custer	29.79	42,883.42	11.02	1,620.42
Davison	33.58	40,754.84	11.95	2,900.42
Day	21.42	35,252.32	14.96	966.32
Deuel	6.47	41,027.05	9.80	700.37
Dewey	6.26	29,018.05	28.47	759.68
Douglas	9.16	37,780.53	12.25	501.05
Edmunds	9.95	42,557.42	11.26	669.63
Fall River	27.16	34,455.47	15.26	1315.79
Faulk	7.79	37,505.05	12.56	384.00
Grant	23.21	41,414.11	9.97	1,254.32
Gregory	14.79	29,423.26	18.78	723.00
Haakon	4.05	37,711.05	12.06	337.37
Hamlin	8.74	42,841.47	10.66	786.42
Hand	9.16	39,506.63	10.56	575.58
Hanson	5.79	46,690.63	9.41	518.53
Harding	2.47	35,922.89	13.27	227.32
Hughes	104.89	50,532.42	9.91	2,927.84
Hutchinson	13.42	38,143.26	12.16	1,120.53
Hyde	5.63	38,027.74	12.33	232.26
Jackson	5.32	27,982.11	30.75	420.26
Jerauld	6.63	37,255.47	13.63	348.79
Jones	4.11	35,868.05	13.72	180.11
Kingsbury	10.16	40,054.53	9.77	855.21
Lake	14.74	42,858.11	10.47	1,767.90
Lawrence	61.16	38,855.68	13.05	3,807.58
Lincoln	22.95	64,288.58	4.51	5,370.79
Lyman	18.42	33,327.53	21.60	552.26
Marshall	6.37	38,048.26	12.52	720.63
McCook	10.84	43,111.26	9.76	859.16
McPherson	7.05	30,467.37	15.35	401.00
Meade	63.58	44,720.05	10.35	3,878.26
Mellette	3.95	27,075.84	32.48	279.42
Miner	4.84	36,984.84	12.06	378.68
Minnehaha	353.21	48,054.37	9.74	24,749.32
Moody	10.68	43,968.21	10.04	1,062.84
Pennington	337.79	42,919.42	13.43	15,432.26
Perkins	13.53	32,988.74	15.15	528.74
Potter	7.95	39,537.84	11.15	412.16
Roberts	16.32	34,492.37	19.67	1,526.11
Sanborn	9.89	38,997.47	13.05	413.05
Oglala Lakota	19.37	24,610.68	43.54	1,472.63
Spink	12.63	38,332.11	12.66	1,101.32
Stanley	11.05	48,299.53	9.15	523.95
Sully	3.47	44,233.63	8.66	239.84
Todd	11.95	23,713.84	41.91	1,102.79
Tripp	15.21	34,837.95	18.42	958.58
Turner	9.58	43,770.89	9.31	1,377.63
Union	16.47	56,129.68	6.64	2,260.11
Walworth	11.11	34,310.42	16.17	933.79
Yankton	28.68	41,454.42	11.94	3,433.47
Ziebach	2.32	23,472.53	46.33	357.42

a Defined as percentage of population with incomes at or below 200% of the Federal Poverty Level.

We summarized the characteristics that pertain to the identified clusters and predictors and the number of participants for 2016 for the 5 clusters (Table 2). Cluster 1 contains the 3 counties with the largest populations, Minnehaha, Pennington, and Hughes counties. This cluster had the smallest average poverty percentage and the highest median income. Cluster 2 had the highest poverty percentage, an average of almost 25%. It also had the lowest median income of the clusters. The overall average number of participants for this cluster was the second-largest even though its average population size was the third largest. Cluster 3 was very similar to Cluster 1 in regard to poverty percentage and median income but had a much smaller population than Cluster 1. Cluster 4’s predictors were in the middle of the other clusters. It had the third-largest poverty percentage and median income of the clusters and the second-smallest population. It contains the second-largest number of counties with 19 in the cluster. Cluster 5 has the smallest average population at only 500. It also had the second-highest number of people living in poverty as indicated by the higher poverty percentage and lower median income than the other clusters. In addition, it had the smallest number of participants, an average of 7 participants in the last 20 years, and the largest number of counties (N = 30), fewer than half of the 66 counties in South Dakota.

**Table 2:** Average of Predictors, All Women Count! Program Participants and Number of Counties for Each Cluster^a^, 2017–2021

Cluster^a^ No.	Average Population	Average Poverty Percentage^b^	Average of US Census Median County Income	No. of 2016 Participants	No. of Counties
**5**	550.71	16.65%	36,917.73	6.14	30
**4**	1,272.55	14.76%	38,828.68	13.49	19
**2**	2,395.26	24.93%	33,814.49	50.42	5
**3**	2,876.77	12.90%	42,656.58	30.56	9
**1**	14,493.21	11.09%	47,420.31	370.76	3

a A cluster is a group of counties with similar sociodemographic characteristics (population, percentage of population with incomes at 200% or below the Federal Poverty Level, median income).

b Defined as the percentage of the population with an annual income at or below 200% of the Federal Poverty Level.

Analysis of forecasts over the next 5 years shows all 5 clusters with an increase in participants (Table 3). Cluster 2 is the only cluster with an expected decrease for a year, occurring in 2018. Cluster 1 is forecasted to have more than 1,000 participants in 2021. Individual county forecasts identified only 6 counties with an expected decrease in the number of participants, one county staying flat, and the rest of the counties with an increased number of participants. Sixteen counties had an observed decrease in participants over the last 5 years, but our model predicted them to have increased participation in the future.

**Table 3:** Forecasted Average of the Number of Particicapnts in the All Women Count! Program for Each County Cluster^a^, 2017–2021

Cluster^a^, No.	2017	2018	2019	2020	2021
**1**	374.18	529.31	707.60	898.70	1,098.41
**2**	41.80	40.67	42.57	43.34	44.66
**3**	34.46	40.38	46.54	52.96	59.65
**4**	13.48	15.83	18.36	20.97	23.69
**5**	5.67	6.69	7.93	9.18	10.51

a A cluster is a group of counties with similar sociodemographic characteristics (population, percentage of population with annual income at or below 200% of the Federal Poverty Level, median income). These calculations are obtained under the assumption that all circumstances stay the same (eg, health care coverage, insurance coverage) over the next 5 years.

**Figure 2:**
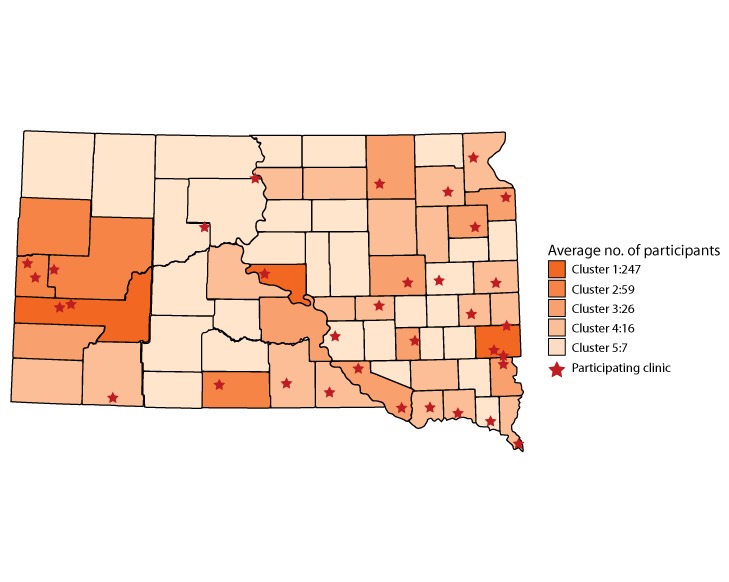
County clusters (groups of counties with similar sociodemographic characteristics [population, percentage of population with annual incomes at or below 200% of the Federal Poverty Level, median income] and AWC! participation) and the 20-year (1997–2016) average annual number of participants in the All Women Count! (AWC!) program in those counties. Red stars indicate that a clinic in that county participated in the AWC! program.

Only two-thirds of clinics in these counties participated for all 10 years. Geographic patterns in county clusters varied (Figure 2). Most of South Dakota’s population is concentrated in the eastern and western parts of the state with the central part sparsely populated. Cluster 1 contained the 2 largest counties on the east and west sides of the state. The counties in cluster 4 appear in groups of 2 to 3, mostly on the eastern side of the state. Most of the low population counties of the central and northwestern parts of the state belong to cluster 5 with the widest scatter. Also, most of the counties in cluster 5 do not have a participating clinic in their county.

**Table:** 

Cluster	Counties in Cluster	Average Annual No. Participants
1	Hughes, Minnehaha, Pennington	265
2	Butte, Lawrence, Meade, Oglala Lakota, Todd	42
3	Beadle, Brown, Charles Mix, Codington, Custer, Davison, Grant, Lincoln, Lyman	31
4	Bon Homme, Brookings, Buffalo, Clark, Day, Edmunds, Fall River, Gregory, Hutchinson, Jerauld, Lake, Moody, Roberts, Spink, Stanley, Tripp, Union, Walworth, Yankton	15
5	Aurora, Bennett, Brule, Campbell, Clay, Corson, Deuel, Dewey, Douglas, Faulk, Haakon, Hamlin, Hand, Hanson, Harding, Hyde, Jackson, Jones, Kingsbury, Marshall, McCook, McPherson, Mellette, Miner, Perkins, Potter, Sanborn, Sully, Turner, Ziebach	7

Our forecast of the average trend in AWC! participation for the identified clusters for the next 5 years, 2017 through 2022, (Table 3) showed that, if all the circumstances stay the same (eg, insurance coverage, policy, advertisement of the AWC! program) participation on average will increase at the cluster level. At the individual county level, forecasts showed that participation in some will increase and will decrease in others. The MSE of training data for years 1997 through 2013 for the state was 21.54, and for the validation data for years 2014 through 2016 was 43.80. The increased test MSE was expected because our training data contained only the first year of decrease from 2012 through 2013. However, when building the final forecasting model, all 20-year data were used; therefore, we expect the error rate on the forecasts to be less than the test MSE reported above.

## Discussion

Our data contained only AWC! screening results. However, 2016 Behavioral Risk Factor Surveillance System (BRFSS) data (21) and Small Area Health Insurance Estimates (SAHIE) (22) data can be used to provide a general context to the AWC! program participation rate. BRFSS results showed that 68% of women in South Dakota aged 40 or older received a mammogram in 2015 and 2016. This is approximately 139,806 women. Of these, we estimated that about 1,926 women aged 40 to 64, about 2.17%, used the AWC! program during those 2 years. Based on the estimates provided in SAHIE data, this is approximately 33% of eligible women in South Dakota.

An exploratory analysis showed an initial increase followed by a decrease in the number of AWC! participants. This may be related to the termination of the WISEWOMEN program, a heart disease screening program that worked in conjunction with AWC! to perform mammography screenings, and the implementation of the Affordable Care Act (ACA). ACA led to an increase in the diagnosis of early-stage cancers, specifically colon and breast cancers, because of an increase in affordability and accessibility of cancer screening (23). Analysis of the effects of these programs or other possible factors on participation needs to be addressed in future work. For example, cluster 5 had a large poverty percentage but low AWC! participation, which may need additional analysis to determine why eligible women were not using the program. Cluster 1 had increased participation for 2016, and further analysis is needed to determine why. Likewise, counties with a large proportion of eligible women screened should be studied to determine factors that possibly contributed to this success. Finally, the model identifies the counties with increasing and decreasing expected participation.

Most forecasting articles we found were on drug use and prescription drug spending. Most of these carried out linear regression analyses. One performed linear regression analysis to aggregate sales data and forecast expected drug expenditures for a hospital (24). Four years of data were used to make predictions for the next 2 years. Similarly, we found another article forecasting resources for US Army health care (25). That study used ordinary least squares estimation, ridge regression, and robust regression and concluded that, although all the models produced nearly the same estimates, ordinary least squares was desirable because it had the simplest interpretations. We considered linear regression for our data. It was, however, too difficult to analyze 66 individual forecasting models for South Dakota counties. Moreover, a forecast for South Dakota as a whole did not provide enough granularity.

In contrast, a mixture of Gaussian regression time series models allowed us to identify, group, and fit models for groups of similar counties. Clustering, as opposed to evaluating single counties, enabled us to use more data when creating forecasts. In our study, we created 5 models from the available data set, as opposed to 66 models by county. If necessary, we can still obtain individual forecasts for each county for more granular analysis.

Several individual county forecasts displayed counter-intuitive trends. Some of these trends may be attributed to an expected increase in forecasted predictors, such as population or median income. This result may also be caused by other predictors, such as advertisement budget, participation through the WISEWOMAN program, or the start of ACA. Future work investigating travel time to the mammography clinic and how that affects participation could also be conducted. Forecasting efforts would also benefit from more comprehensive data sets that include data related to other state programs (eg, Indian Health Services) to show the total number of women participating in screening programs. Forecasting projects for similar cancer screening programs in other states will help both to validate our methodology and to improve models for screening programs in general.

The identification of county clusters may assist the South Dakota Department of Health to allocate and manage resources more effectively. The results of our study indicate which counties may see an increase in number of AWC! participants. Hence resource allocation decisions could be tailored on the basis of need, which would lead ultimately to an increase in breast cancer screening rates and early detection of breast cancer. In addition, our results may help the South Dakota Department of Health determine which counties would benefit more from a mobile mammography unit, which would reduce barriers to mammography screening, reach underserved populations, and thus address breast cancer disparities in rural areas. This aligns with the goals and objectives of AWC! and the South Dakota Comprehensive Cancer Control State Plan (26).

Our study identified clusters and forecasted the trend in AWC! participation for the next 5 years. According to our model, the number of participants will increase in some counties and decrease in others. Forecasting is a complex analysis; though our analysis was limited by the number of predictors, this is the first forecasting study among cancer screening programs. Our work provides information for AWC! managers engaged in budgeting and planning strategies to increase screening rates among underserved women in South Dakota.

## Post-Test Information

To obtain credit, you should first read the journal article. After reading the article, you should be able to answer the following, related, multiple-choice questions. To complete the questions (with a minimum 75% passing score) and earn continuing medical education (CME) credit, please go to http://www.medscape.org/journal/pcd. Credit cannot be obtained for tests completed on paper, although you may use the worksheet below to keep a record of your answers.

You must be a registered user on http://www.medscape.org. If you are not registered on http://www.medscape.org, please click on the “Register” link on the right hand side of the website.

Only one answer is correct for each question. Once you successfully answer all post-test questions, you will be able to view and/or print your certificate. For questions regarding this activity, contact the accredited provider, CME@medscape.net. For technical assistance, contact CME@medscape.net. American Medical Association’s Physician’s Recognition Award (AMA PRA) credits are accepted in the US as evidence of participation in CME activities. For further information on this award, please go to https://www.ama-assn.org. The AMA has determined that physicians not licensed in the US who participate in this CME activity are eligible for *AMA PRA Category 1 Credits™*. Through agreements that the AMA has made with agencies in some countries, AMA PRA credit may be acceptable as evidence of participation in CME activities. If you are not licensed in the US, please complete the questions online, print the AMA PRA CME credit certificate, and present it to your national medical association for review.

## Post-Test Questions

### Study Title: Forecasting Participants in the All Women Count! Mammography Program

#### CME Questions

1. You are advising a rural area regarding implementation of a no-cost mammography screening program for qualified women. According to the study and modeling analysis by Holzhauser and colleagues, which of the following statements about present usage of mammography screening in the AWC! program and clusters of counties with similar socioeconomic characteristics is correct?
  - The number of AWC! participants increased steadily from 1997 through 2016
  - Eight clusters of South Dakota counties had similar predictor variables and number of participants
  - Cluster 1 had 3 counties with the largest populations, the smallest average poverty percentage, and the highest median income
  - The cluster with the smallest average population had the smallest number of counties
2. According to the study and modeling analysis by Holzhauser and colleagues, which of the following statements about future usage of mammography screening in the AWC! Program is correct?
  - The mixture model showed that the number of participants will increase in all counties
  - Analysis of forecasts over the next 5 years shows an increase in participants in 3 of 5 clusters
  - If all circumstances stay the same (eg, insurance coverage, policy, advertisement of the AWC! program), participation on average will remain the same at the cluster level
  - Although 16 counties had a decrease in participants over the last 5 years, the model predicted they would have increased participation in the future
3. According to the study and modeling analysis by Holzhauser and colleagues, which of the following statements about public health implications of findings regarding present and forecasted usage of mammography screening in the AWC! program is correct?
  - Decrease in number of AWC! participants may be related to implementation of the WISEWOMEN program
  - Implementation of the Affordable Care Act (ACA) was unlikely to affect number of AWC! participants
  - Counterintuitive trends in individual county forecasts may be attributed to an expected increase in forecasted predictors (eg, population, median income, or advertising budget)
  - Identification of county clusters is unlikely to facilitate effective resource allocation and management
